# Blockade of CCR3 retains the neutrophils, preserving their survival during healing after myocardial infarction

**DOI:** 10.15190/d.2015.37

**Published:** 2015-06-30

**Authors:** Adelina Curaj, Mareike Staudt, Roxana Fatu, Andreas O. Kraaijeveld, Joachim Jankowski, Erik A.L. Biessen, Elisa A. Liehn

**Affiliations:** Institute for Molecular Cardiovascular Research (IMCAR), RWTH Aachen University, Germany; "Victor Babes" National Institute of Pathology, Bucharest, Romania; Department of Cardiology and Einthoven Laboratory of Experimental Vascular Medicine, Leiden University Medical Center, The Netherlands; Department of Pathology, Academic University Hospital Maastricht, Maastricht, The Netherlands

**Keywords:** myocardial infarction, CC-chemokine, neutrophils, left anterior descending artery (LAD)

## Abstract

BACKGROUND: Chemokines are critical mediators in controlling and monitoring the healing and ventricular remodeling after myocardial infarction (MI). They proved to be valuable targets for therapeutic measures to reduce the scar formation and to preserve heart function in patients suffering MI. In the present study, the role of CCR3 in myocardial ischemia/reperfusion was established. METHODS AND RESULTS: One week after infarct induction in a mouse coronary ligation model, the functional and morphological parameters of the heart were analyzed. Isolated-heart Langendorff perfusion showed no significantly differences in heart function, infarction size and post infarction angiogenesis after CCR3 blockade. Apoptotic, proliferation signals as well as collagen synthesis were not affected in CCR3 antagonist treated mice. Notably, CCR3 inhibition was accompanied by massive neutrophil infiltration, while leaving the presence of other immune cell subsets in heart unaffected. CONCLUSION: Since neutrophils represents one of the most widely explored therapeutic targets in the treatment of cardiac disease, this study may open a new perspective for a better understanding of the physiology and homeostasis of neutrophils and points out new directions for intervention in acute MI.

## INTRODUCTION

Despite extensive research in the last decades, myocardial infarction (MI) still remains the first cause of death in developed countries. The acute blockage of a coronary artery leads to a complex inflammatory response, to the cellular debris, as well as to start healing processes and restore the functional integrity of the heart as soon as possible^1^. In this regards, the efforts of scientific community are focused on finding new therapeutic strategies based on a better understanding of the molecular and cellular events.

Chemokines are critical mediators in controlling and monitoring all these events and therefore, they have been established not only as predictors of heart injury^2-4^, but also as potential and valuable pharmacological targets^5^. CC chemokines are mostly involved in the recruitment of monocytes and macrophages, while CXC family of chemokines have essential roles in neutrophil, B- and T-cell recruitment^6^. During healing after myocardial infarction, neutrophils were seen to be recruited mainly through CCR1^7^ and CXCR4^8^ and to a lesser extent through CXCR2^9,10^. Inflammatory monocytes are responsive to CCR2^11^ ligands but not to CCR1 and CCR5^12-14^ while later on the inflammatory cascade non-classical monocytes will be recruited via CX3CR1^12^. T cells were reported to exert their regulatory functions and reduce adverse remodeling of the infarcted heart via CCR5^15^. Post infarct neoangiogenesis, which supports the scar formation, depends on the recruitment of endogenous endothelial progenitor cells to ischemic myocardium by CCR3- and CXCR4-mediated interactions^16^.

Although CCR3 is expressed by variety of immune cells, such as eosinophils, basophils, mast cells, lymphocytes or monocytes and macrophages^17^, which all are relevant to MI, and as it bind a wide range of ligands, such as CCL5, CCL2, CCL7, CCL11, CCL13, CCL15, CCL24 or CCL26^18^, its contribution to post-MI inflammation has not yet been addressed. In this study, we have dissected CCR3’s role in healing after MI, using a pharmacological CCR3-antagonist in a mouse model of myocardial ischemia/reperfusion.

## MATERIALS AND METHODS

### Murine model of myocardial ischemia/reperfusion (I/R)

Eight weeks-old male C57Bl/6J mice underwent coronary occlusion and reperfusion as described^19^. Briefly, mice were intubated under general anesthesia and positive pressure ventilation was maintained using a mouse ventilator. Hearts were exposed by left thoracotomy and MI was produced by suture occlusion of the left anterior descending artery (LAD) over a silicon tube. The ligature was removed after 30 min to permit reperfusion. The ribs, muscle layer and skin incision were closed, and morphine (Buprenorphine; 0.1 mg/kg) was administrated until full recovery. The mice were treated orally with a CCR3 antagonist (20 mg/kg, GW-766994, GlaxoSmithKline) or vehicle, one day before MI and 3 times per week. All animal experiments and study protocols were approved by local authorities, complying with German animal protection laws (AZ 50.203.2-AC 37, 26/05).).**

### Langendorff perdusion

Seven days after MI, heart function was analyzed using a Langendorff apparatus with Isoheart software (Hugo Sachs Elektronik-Harvard Apparatus) under constant perfusion pressure and electrical stimulation (600/min constant heart rate). Coronary flow, left ventricular developed pressure (LVDP), increase (dP/dtmax) and decrease (dP/dtmin) in LV pressure were measured without and with dobutamine. Finally, hearts were embedded in paraffin and cut into 5 mm serial slices.

### Morphometrical analysis

Ten serial sections per mouse (each 400 µm apart, up to the mitral valve) were stained using Gomori’s 1-step trichrome stain, as previously described^8,19^. The infarcted area was determined in all ten sections and expressed as percentage of total LV area, using Diskus software (Hilgers). Cell content was also analyzed in serial section of hearts after MI. Sections (3 per mouse, 400 mm apart) were stained for neutrophils (specific esterase, Sigma and KC, RnD Systems), macrophages (F4/80, Serotec), lymphocytes (CD3, Serotec), vessels (CD31, Santa Cruz), and Collagen (Gomori’s 1-step trichrome stain). Cells were numbered in six different fields inside the infarcted area in each section and expressed as cells/mm² using DISKUS (Hilgers, Germany). Apoptotic cell (In Situ Cell Death Detection Kit, Roche) or proliferation (anti-Ki-67, DAKO) indexes were expressed as percentage of positive cells from total nuclei (DAPI).

### Statistical analysis

Statistical analysis was performed with Prism5 software (GraphPad Prism) using Student t test. P-values of <0.05 were considered significant

## RESULTS

One week after ischemia/reperfusion induction in mock or CCR3 antagonists (GW-766994) treated mice, the functional parameters of the heart were recorded using isolated Langendorff heart perfusion. Interestingly, GW-766994 treatment did not affect or improve significantly the heart function or the infarction size, as shown in **Table 1**. Moreover, we were unable to detect any significant differences in the degree of neo-angiogenesis, as well as in coronary flow compared with control group (**Table 1**).

**Table 1:** Morphometrical and functional parameters after anti-CCR3 treatment. LVDP: left ventricular developed pressure; dPdtmax: maximum time-derived pressure over time; dPdtmin: minimum time-derived pressure over time; n.s.: not significant.

	Control n=6	Anti-CCR3 n=5	P Value
*Infarction size* (% of lect ventricle)	5.08 ± 0.88	3.62 ± 1.25	n.s.
*Neo-angiogenesis* (CD31 positive rings/mm^2^)	149.80 ± 11.70	133.60 ± 28.51	n.s.
*LVDP* (mmHg)	23.92 ± 3.45	20.20 ± 4.212	n.s.
*dPdtmax* (mmHg/s)	1477 ± 153	1594 ± 442	n.s.
dPdtmin (mmHg/s)	-956 ± 108	-938 ± 117	n.s.
Coronary flow (ml/min)	1.60 ± 0.20	1.95 ± 0.15	n.s.
Collagen (% from infarcted area)	18.42 ± 2.5	18.35 ± 1.8	n.s.

**Figure 1:**
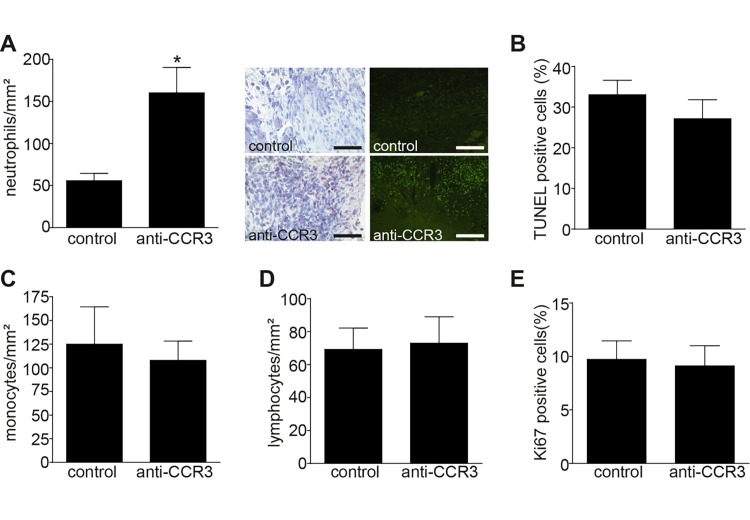
(A) Neutrophils could be traced inside the infarcted area one week after MI, as shown by representative images of specific esterase (middle panel, red) and KC staining (right panel, green). (B) TUNEL positive signals, (C) monocytes, (D) lymphocytes and (E) Ki67 positive cells do not differ between the groups after anti-CCR treatment.*p<0.05. Scale bars 50 µm.

Notably, one week after MI, we observed massive neutrophil infiltration in the GW-766994 treated group, while isolated neutrophils were barely observed in control mice at this time point (**Figure 1** [A]). Since apoptotic signals were not amplified (**Figure 1** [B]), this suggests that neutrophils are retained within the infarcted areas unable to migrate or to die. Other immune subsets, such as monocytes/macrophages (**Figure 1** [C]) and lymphocytes (**Figure 1** [D]) were not affected by the GW-766994 treatment. The proliferation (**Figure 1** [E]) and the collagen deposition (**Table 1**) inside the infarcted area remain comparable between the groups.

## Discussion

In this study, we were able to demonstrate that the neutrophils trafficking into the infarcted area was significantly affected by the anti-CCR3 treatment, while morphological and functional parameters of the heart after MI were not changed. These findings are not only novel, but also very surprising, since the negative remodeling after MI was always connected with the neutrophil infiltration. Neutrophils are the first cells to be recruited to the infarction site, releasing inflammatory mediators, proteolytic enzymes and reactive oxygen species, which directly injure the surrounding living cells.

Neutrophil depletion in animal models led to a marked decrease in infarct size and therefore, impairing the neutrophil infiltration into the infarcted area represents one of the most discussed therapeutic strategies inside the cardiology community. However, the present study confirms other previous data^10^ which suggest that in some cases, impairing the neutrophil recruitment do not have any influence on later healing and repairing processes after MI. A specific and selective therapeutic intervention by blocking only IL-1 pro-inflammatory signals was also proposed as a more valuable and feasible alternative^20^.

On the other hand, the fast disappearance of the neutrophils from the infarcted area was always attributed to their programmed death. However, the triggers and the mechanisms of these processes are far from being understood. Mice lacking CCR3 showed defective eosinophil and mast cells trafficking, accumulating into lung parenchyma, being unable to migrate outside the subendothelial space^21^. In our model, the neutrophils are trapped inside the infarcted area and are unable to activate the apoptotic pathways, which demonstrates the crucial role of CCR3 in the fate of neutrophils during the healing after MI. Although this finding is interesting and surprising, further investigations are necessary to elucidate the entire mechanisms and connections until we will be able to adjust the current therapeutic strategies for the benefit of patients.

The last surprising finding of our study is the lack of effect on angiogenesis after MI. There are reports demonstrating the role of CCR3 in the recruitment of CD34 positive progenitor cells from bone marrow to ischemic, but not to normal myocardium^16^. We did not find any significant differences between the groups in the angiogenesis and coronary flow after MI, probably due to the sufficient compensatory mechanisms^22^.

In conclusion, despite the lack of effects on heart function of the CCR3’s pharmacological blockade, this study opens a new perspective in better understanding the physiology and homeostasis of neutrophils, which have the crucial role in initiation and progression of the healing and scar formation after MI. Only knowing their precise role, fate and the interaction with all other involved players, we will be able to propose and develop new therapeutic strategies to reduce the complications after MI and to increase the life quality of the patients.
